# Multi-Omics Analysis Reveals Mechanisms of Strong Phosphorus Adaptation in Tea Plant Roots

**DOI:** 10.3390/ijms241512431

**Published:** 2023-08-04

**Authors:** Xiaomei Liu, Jing Tian, Guodao Liu, Lili Sun

**Affiliations:** 1 College of Tropical Crops, Hainan University, Haikou 570228, China; liuxiaomeitx@yeah.net; 2 Haixia Institute of Science and Technology, Fujian Agriculture and Forestry University, Fuzhou 350002, China; tianjing9808@163.com; 3 Institute of Tropical Crops Genetic Resources, Chinese Academy of Tropical Agriculture Sciences, Haikou 570228, China; liuguodao2008@163.com

**Keywords:** tea plant, phosphorus, root growth, multi-omics

## Abstract

Low phosphorus (P) is a major limiting factor for plant growth in acid soils, which are preferred by tea plants. This study aims to investigate the unique mechanisms of tea plant roots adaptation to low-P conditions. Tea plant roots were harvested for multi-omics analysis after being treated with 0 µmol·L^−1^ P (0P) and 250 µmol·L^−1^ P (250P) for 30 days. Under 250P conditions, root elongation was significantly inhibited, and the density of lateral roots was dramatically increased. This suggests that 250P may inhibit the elongation of tea plant roots. Moreover, the P concentration in roots was about 4.58 times higher than that under 0P, indicating that 250P may cause P toxicity in tea plant roots. Contrary to common plants, the expression of *CsPT1/2* in tea plant roots was significantly increased by four times at 250P, which indicated that tea plant roots suffering from P toxicity might be due to the excessive expression of phosphate uptake-responsible genes under 250P conditions. Additionally, 94.80% of P-containing metabolites accumulated due to 250P stimulation, most of which were energy-associated metabolites, including lipids, nucleotides, and sugars. Especially the ratio of AMP/ATP and the expression of energy sensor *CsSnRKs* were inhibited by P application. Therefore, under 250P conditions, P over-accumulation due to the excessive expression of *CsPT1/2* may inhibit energy metabolism and thus the growth of tea plant roots.

## 1. Introduction

As an essential macronutrient for plant growth and development, P serves not only as a structural element of cell membranes and genetic materials but also as a constituent element of energy materials like ATP and phosphate esters [1]. However, in acidic soils, P primarily exists in organic and insoluble inorganic forms that are difficult for plants to absorb and for the soil to move [2]. Low P levels have become one of the primary limiting factors for crop growth in acidic soil areas. Furthermore, almost half of the world’s arable soil is acidic [3], making it vital to explore the biological mechanisms of plant adaptation to low-P environments.

In order to adapt to low-P conditions, plants have developed various strategies to improve the efficiency of P absorption and utilization. These strategies include changing root architecture or morphology and the expression of P transporters in roots to promote P uptake, secreting organic acids or phosphatases to increase P availability in soils, and regulating key genes such as *SPX* to coordinate intracellular P homeostasis [4,5]. Among these strategies, the P signaling network has been extensively and deeply studied. *SPX*, a key transcription factor in the P signaling network, is usually upregulated by low P to enhance its expression [6]. Overexpression of *PvSPX1* in *Phaseolus vulgaris* significantly upregulates the expression of downstream P transport key genes, including *PvPHT2* and *PvPT1*, thereby increasing P concentration in transgenic hairy roots [7]. However, the expression of *PHO2*, which encodes the E2 ubiquitin ligase, is usually inhibited by low P and negatively regulates the P signaling network genes [8]. The transcription level of *OsSPX1* is increased in the rice *pho2* mutant, indicating that upstream *OsPHO2* may repress the transcriptional expression of *OsSPX1* [9]. Downstream genes in the P signaling network that encode phosphate transporters are also important, and currently, four major families have been identified, with the PT1 family having the largest number of members responsible for phosphate absorption and transport [10,11,12]. In Arabidopsis, all PT1 family members except *PT1;6* are significantly induced by low P in roots [13]. However, P uptake by roots is significantly reduced under low-P conditions when the *Pht1;4* gene, one of the key high-affinity phosphate transporters in Arabidopsis, is mutated [14].

Tea (*Camellia sinensis*) is widely consumed due to its abundant health metabolites, and it can only be cultivated in acidic soils, where Aluminum (Al) toxicity and P deficiency are major factors limiting plant growth [3]. Our previous study found that Al is an essential element for tea plant root growth [15]. Therefore, tea plants may have a unique adaptation mechanism to low-P conditions. Previous studies have found that tea plants exhibit strong P utilization efficiency under P deficiency conditions. Compared to tea plants without P addition, they showed unchanged root surface area, reduced P uptake rate, and ultimately exhibited nearly 3-fold higher P utilization efficiency, which was significantly higher than that of *Phaseolus vulgaris* control plants adapted to a low P environment [16]. In terms of P uptake, Lin et al. [17] suggested that tea plants increase the synthesis and secretion of organic acids under low-P conditions, which contributes to improving P availability in soil. Additionally, tea plants may avoid photooxidative damage to the leaves by mechanisms such as blocking the photophosphorylation transport chain to reduce ATP content or reducing the activity of antioxidant enzymes [18,19]. The omics approach can comprehensively analyze the adaptation mechanisms of plants to the environment and effectively screen key influencing factors [20]. Previous studies have also used multi-omics methods to analyze different omics profiles of tea plants under low-P conditions [21,22]. However, these studies mainly focused on the effect of P deficiency on the leaf quality of tea plants, including changes in major quality metabolites such as flavonoids and amino acids.

In this study, we investigated the effects of P on the growth and development of tea plant roots by treating plants with and without P and analyzing the changes in nutrient absorption by roots using ionomics. Additionally, we used transcriptomics and metabolomics to identify the key genes and metabolites affected by P in tea plant roots. Through this comprehensive approach, we aimed to elucidate the physiological and molecular mechanisms underlying the adaptation of tea plants to low-P environments in acidic soil.

## 2. Results

### 2.1. 250P Inhibited Tea Plant Root Growth

**Figure 1:**
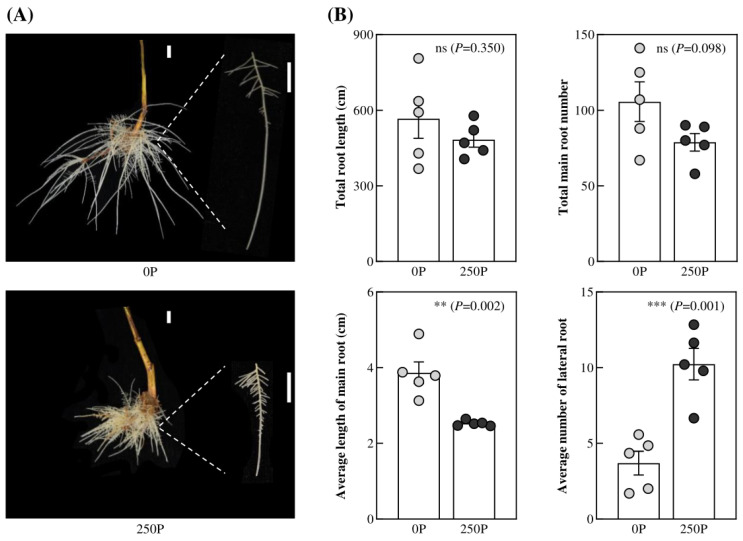
Root growth of tea plants affected by P treatments. (**A**) Phenotypes of tea plant roots. The scale bars indicate 1 cm. (**B**) Total root length, total main root number, average length of main roots, and average lateral root number of tea plant roots. 0P, 0 μmol·L^−1^ P; 250P, 250 μmol·L^−1^ P. Each treatment had five biological replicates. Asterisks indicate statistical differences. ns, no statistical differences; **, significant differences at 0.001 < *p* ≤ 0.01; ***, significant differences at *p* ≤ 0.001.

To investigate the effect of P on tea plant root growth, we conducted a hydroponic experiment using P-deficient and P-supplemented solutions. After 30 d, we analyzed the total root length, total root number, average primary root length, and average lateral root density of tea plants. Our results revealed that P had a significant impact on tea plant root growth and development. In contrast to most plants, tea plant roots appeared white and healthy under 0P, while they turned yellow and showed a short and dense phenotype under 250P (Figure 1A). Specifically, the average primary root length of tea plants was only 2.526 cm, and the average lateral root density was 10.228 under 250P conditions. Compared to 0P, the average primary root length of tea plants decreased significantly by 34.72% under 250P, whereas the average lateral root density increased by 1.76-fold (Figure 1B). These findings indicated that 250P treatment inhibited primary root elongation and promoted lateral root development in tea plants. Interestingly, we found no significant difference in the total root length and total root number of tea plants between the 0P and 250P treatments (Figure 1B). This result is consistent with previous studies [16], which suggest that increasing the root surface area of tea plants under P deficiency does not enhance P uptake. Overall, our results suggest that P has a distinct impact on tea plant root growth, inhibiting primary root growth and promoting lateral root development, leading to a blunted root phenotype.

### 2.2. 250P-Increased Expression of PT and Over-Accumulation of P in Tea Plant Roots

**Figure 2:**
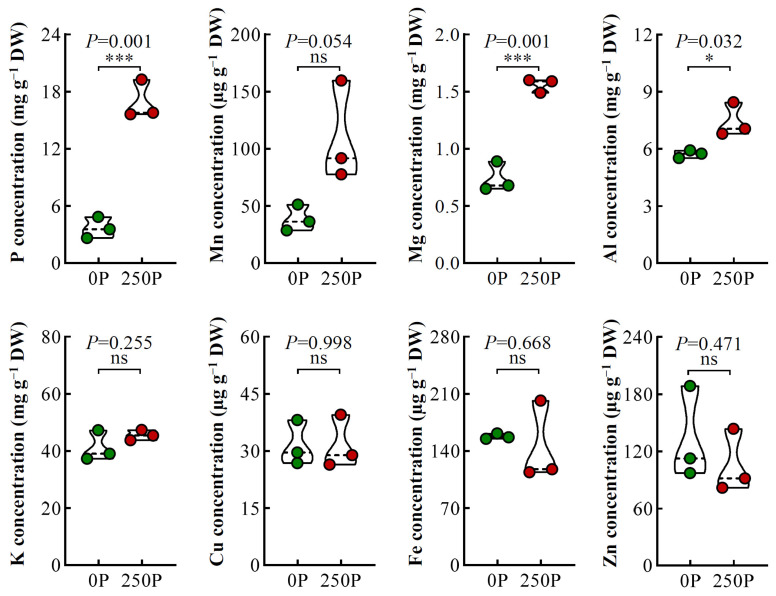
Concentrations of mineral elements in tea plant roots are affected by P treatments. 0P, 0 μmol·L^−1^ P; 250P, 250 μmol·L^−1^ P. Three biological replicates were performed for each treatment. Asterisks indicate statistical differences. ns, no statistical differences; *, significant differences at 0.01 < *p* ≤ 0.05; ***, significant differences at *p* ≤ 0.001.

To explore the effects of P treatments on the nutrient content of tea plant roots, an ionome analysis was performed. P treatment had a significant effect on the nutrient status of tea plant roots, particularly on P, Mn, Mg, and Al (Figure 2). The P concentration in tea plant roots was 3.68 mg·g^−1^ under 0P and significantly increased by 3.58-fold at 250P (Figure 2), indicating a possible luxury uptake of P in tea plant roots under high-P conditions. Tea plant roots exhibited increased absorption of Al, an essential nutrient for their growth and development [15], with an approximately 29.62% increase under 250P. Additionally, P significantly enhanced the concentration of Mg in tea plant roots, with approximately twice as much Mg at 250P as at 0P (Figure 2). Overall, the results indicated that excessive P was absorbed by tea plant roots in the presence of P, leading to increased Al and Mg concentrations in the roots.

**Figure 3:**
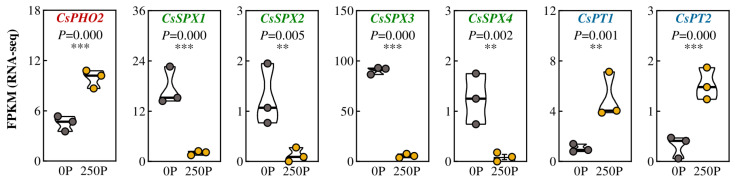
Expression of key genes associated with the P signal network in tea plant roots affected by P treatments. 0P, 0 μmol·L^−1^ P; 250P, 250 μmol·L^−1^ P. Three biological replicates were performed for each treatment. Asterisks indicate statistical differences. **, significant differences at 0.001 < *p* ≤ 0.01; ***, significant differences at *p* ≤ 0.001.

Due to the joint regulation of P absorption by upstream and downstream P signals [23], we analyzed the transcriptome results of tea plants to identify the significantly differentially expressed P signal genes. In the root of tea plants, we identified five genes containing the SPX domain, one of which encoded ubiquitin protease, and the other four belonged to the SPX family, named *CsPHO2* and *CsSPX1/2/3/4*, respectively (Figure 3). CsPHO2 was in the same evolutionary branch as PHO2 from Arabidopsis, rice, and soybean and had the highest homology with soybean GmPHO2a/b (Figure S1A). Gene expression analysis revealed that *CsPHO2* was significantly upregulated by more than 2-fold under 250P (Figure 3 and Figure S2A). CsSPX1/2 were in the same evolutionary branch as Arabidopsis AtSPX3 and soybean GmSPX1, while CsSPX3/4 showed higher homology with Arabidopsis AtSPX1/2 (Figure S1B). Under 250P, the gene expression of *CsSPX1/2/3/4* was significantly inhibited, decreasing by 88.19%, 90.48%, 97.74%, and 76.80% compared to 0P (Figure 3 and Figure S2A). These findings suggest that, similar to the regulatory network of P signaling in common plants [23], P promotes the expression of *CsPHO2* while repressing the expression of *CsSPX1/2/3/4* in tea plants. The P transporter gene is a crucial factor affecting P absorption [24]. In the transcriptome results, two P transporter genes, *CsPT1/2*, showed significant changes in expression levels in response to P regulation (Figure 3). In the evolutionary tree, CsPT1/2 exhibited high homology with the Arabidopsis key P transport proteins AtPht1.4 and AtPht1.7 (Figure S1C). Surprisingly, the expression of *CsPT1/2* was dramatically enhanced by P stimulation. Under 250P, the expression levels of *CsPT1* and *CsPT2* increased almost four-fold (Figure 3 and Figure S2A). These results suggest that the expression of genes encoding P transporters in tea plant roots is inhibited under P deficiency but promoted in the presence of P, which may contribute to the excessive uptake of P by tea plant roots under 250P conditions.

### 2.3. 250P Reduced Expression of SCPLs and Synthesis of Esterified Catechins

**Figure 4:**
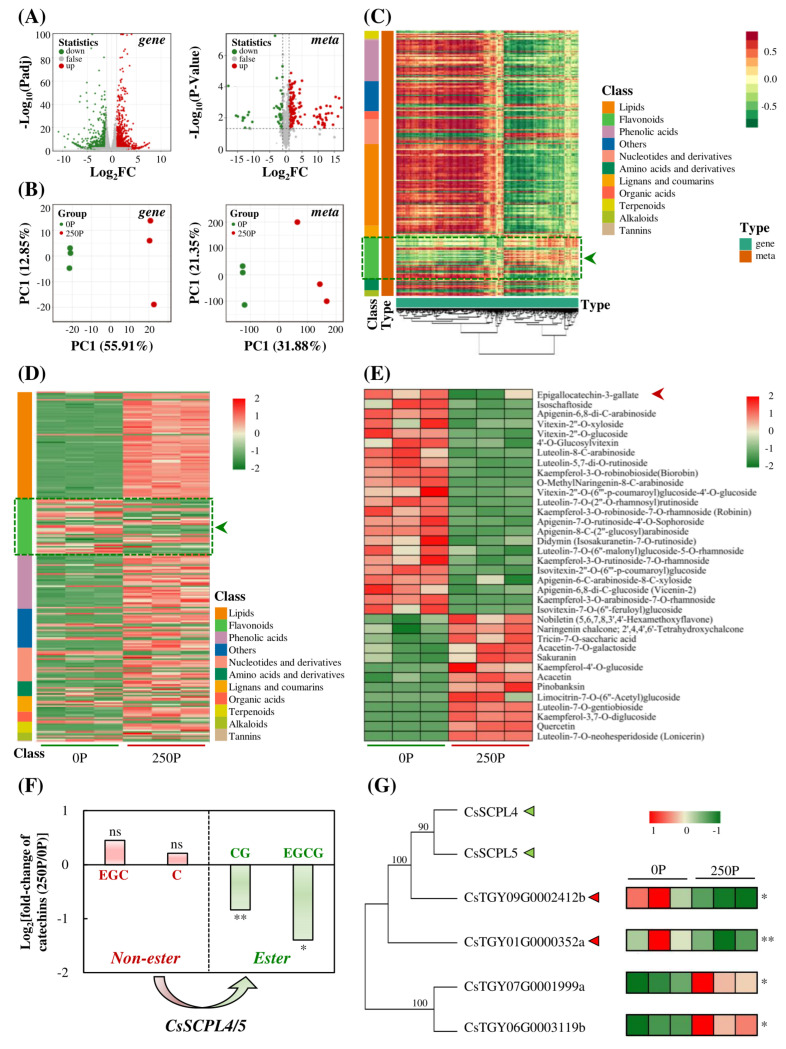
Effects of P on catechins in tea plant roots through transcriptomics and metabolomics. Volcano map (**A**) and PCA (**B**) analysis of differentially expressed genes and metabolites in tea plant roots. Gene, transcriptomic analysis; meta, metabolomics analysis. (**C**) Conjoint correlation heat map combined with transcriptome and metabolome. (**D**) Heat map of differentially changed metabolites. (**E**) Heat map of differentially changed flavonoids. (**F**) Changes in the main catechins in tea plant roots. (**G**) Phylogenetic tree and heat map of differentially expressed genes homologous to *CsSCPL4/5*. The dark green arrow indicates a special group with a different pattern from other groups. The red arrow shows an important metabolite in tea plants. The light green triangle indicates the reported genes responsible for esterification of catechin in tea plants. The red triangle indicates the potential genes for catechin esterification regulated by P. 0P, 0 μmol·L^−1^ P; 250P, 250 μmol·L^−1^ P. Three biological replicates were performed for each treatment. Asterisks indicate statistical differences. ns, no statistical differences; *, significant differences at 0.01 < *p* ≤ 0.05; **, significant differences at 0.001 < *p* ≤ 0.01.

To comprehensively analyze the effect of P on the root growth of tea plants, this study investigated both the transcriptome and metabolome. The transcriptome analysis identified 2651 genes that were significantly differentially expressed under 250P treatment, with 58.32% up-regulated. The metabolome analysis detected a total of 231 differentially characteristic metabolites, with 54.52% enriched and increased under 250P treatment (Figure 4A), indicating a greater number of up-regulated genes and metabolites than down-regulated ones under 250P treatment. Principal component analysis (PCA) of the differential genes in the transcriptome and differential metabolites in the metabolome showed that the samples treated with 0P and 250P were horizontally separated, with the first principal component (PC1) explaining 55.91% and 31.88% of the total variance of the transcriptome and metabolome data, respectively, while the second principal component (PC2) explained 12.85% and 21.35% of the total variance, respectively (Figure 4B). These results suggest that 250P significantly influenced gene expression and metabolite accumulation in tea plant roots.

A joint analysis of the transcriptome and metabolome data revealed a unique trend in the correlation between flavonoid-enriched metabolites and differentially expressed genes, which was opposite to that of other metabolites (Figure 4C). Heat maps were generated for the differential metabolites, and the trend of variation in flavonoid metabolites regulated by P was different from that of other metabolites, with a significant decrease in relative content under 250P treatment (Figure 4D). Of the 36 flavonoids affected by P, the relative content of 23 metabolites, including epigallocatechin-3-gallate (EGCG), decreased significantly under 250P (Figure 4E). These findings suggest that P may inhibit the synthesis of flavonoid metabolites in tea plant roots, including EGCG, an ester catechin. Catechin is the main secondary metabolite in tea plants, and esterified catechins, especially EGCG, are recognized as health-promoting substances that have an important impact on tea quality [25]. In this study, we also analyzed other major catechins. We found that 250P treatment significantly reduced the formation of esterified catechins, including EGCG and catechin gallate (CG), while the precursors of these catechins, EGC and C, accumulated (Figure 4F). These results suggest that 250P may affect the esterification process of catechins. Previous studies have shown that the serine carboxypeptidase-like acyltransferase gene *CsSCPL4/5* plays a key role in regulating the catechin esterification process in tea plants [26]. Four genes encoding serine carboxypeptidase-like acyltransferase were identified from the transcriptome results, and the proteins CsTGY09G0002412b and CsTGY01G0000352a had a close evolutionary relationship with CsSCPL4/5 (Figure 4G), suggesting that they might also have a function in catalyzing catechin esterification. However, the expression of *CsTGY09G0002412b* and *CsTGY01G0000352a* was significantly reduced under the 250P treatment condition (Figure 4G and Figure S2B), indicating that 250P might inhibit the esterification of catechins, such as EGCG, by reducing the expression of esterification genes.

### 2.4. 250P-Decreased Synthesis of AMP/ATP Ratio and Expression of SnRKs

**Figure 5:**
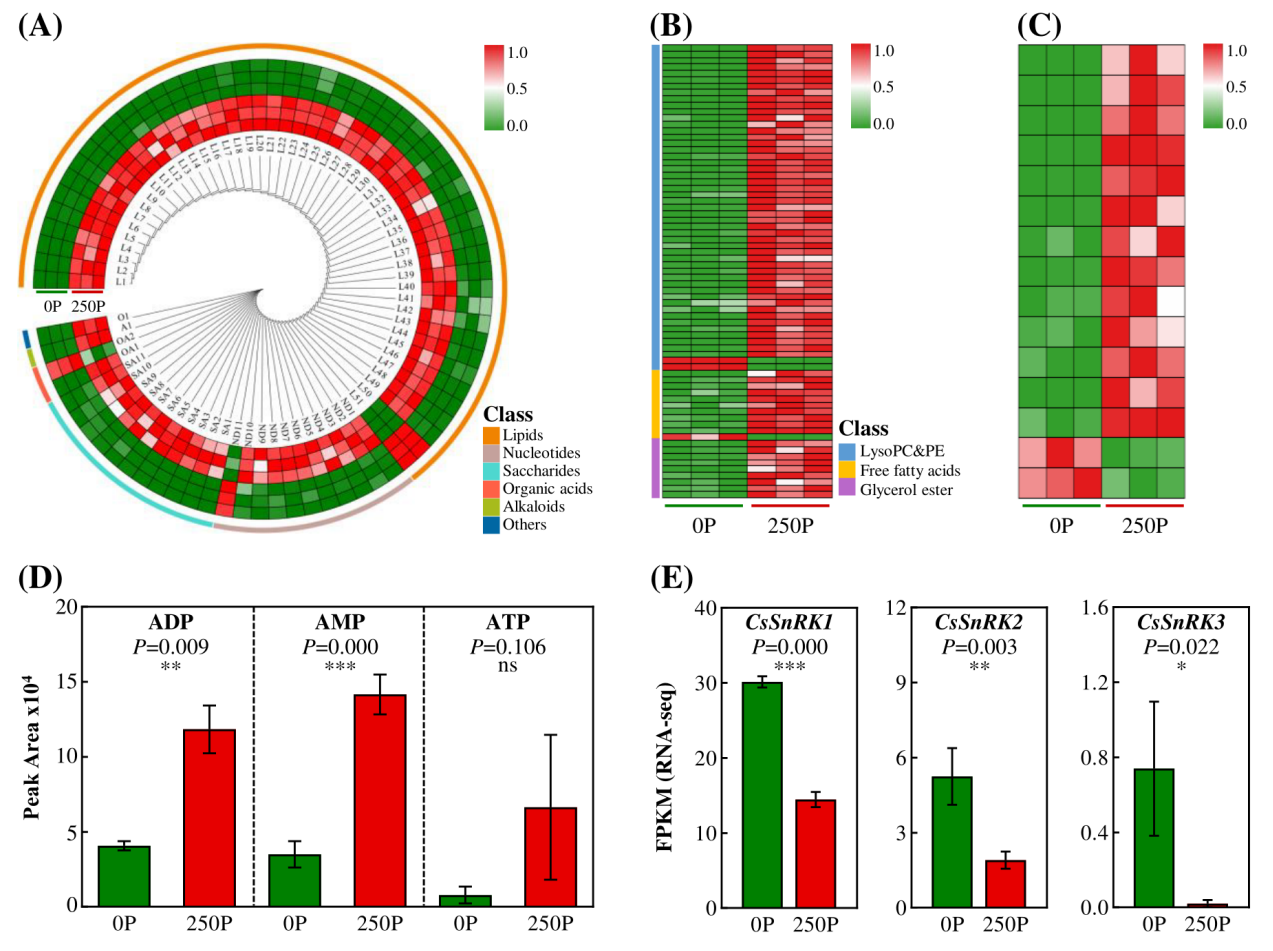
Effects of P on energy metabolism in tea plant roots through transcriptomics and metabolomics. (**A**) Heat map of differentially changed P-containing metabolites. (**B**) Heat map of differentially changed lipid metabolites with or without P. (**C**) Heat map of differentially changed carbohydrate metabolites with or without P. (**D**) Accumulation of ADP, AMP, and ATP metabolites. (**E**) Gene expression of *CsSnRKs*. 0P, 0 μmol·L^−1^ P; 250P, 250 μmol·L^−1^ P. Three biological replicates were performed for each treatment. Asterisks indicate statistical differences. ns, no statistical differences; *, significant differences at 0.01 < *p* ≤ 0.05; **, significant differences at 0.001 < *p* ≤ 0.01; ***, significant differences at *p* ≤ 0.001.

Further analysis of all P-containing metabolites showed that treatment with 250P significantly promoted the accumulation of P-containing metabolites in tea plant roots. Among the 77 P-containing metabolites that showed significant changes, 51 were lipids, 11 were nucleotides, and 11 were sugars (Figure 5A; Table S1). P-containing lipids were the most abundant, mainly including phosphatidylcholine (PC), lysophosphatidylcholine (LPC), and lysophosphatidylethanolamine (LPE). Additionally, P-free lipids also accumulated at 250P, including free fatty acids and glycerol esters (Figure 5B; Table S2). These findings indicate that P promotes the accumulation of lipid metabolites. Moreover, 250P treatment not only increased the levels of lipids but also promoted the synthesis and accumulation of 13 carbohydrate metabolites in tea plant roots, including two P-free carbohydrate metabolites (Figure 5C; Tables S1 and S3). Additionally, the levels of the main adenosine phosphate metabolites, ADP, AMP, and ATP, increased by 2.88, 4.06, and 8.25 times, respectively, under 250P (Figure 5D). These findings demonstrate that P enhances the accumulation of energy metabolites in tea plant roots, including lipids, sugars, and adenosine phosphate.

Interestingly, compared to 0P, 250P treatment effectively decreased the ratio of ADP/ATP and AMP/ATP by 65.42% and 51.73%, respectively (Figure 5D), indicating that tea plant roots were in a high energy state under 250P. In plants, SnRK1 is a crucial energy-sensing kinase [27]. Notably, compared to 0P, 250P treatment significantly reduced the expression of *CsSnRK1/2/3* in tea plants, with decreases of 52.04%, 63.81%, and 97.30%, respectively (Figure 5E and Figure S2C). Therefore, these results suggest that under low-P conditions in acid soils, tea plants regulate the synthesis and accumulation of energy-related metabolites such as lipids, carbohydrates, and adenosine by controlling the expression and activation of the energy-sensing kinase CsSnRKs, ultimately impacting their adaptability to the environment.

## 3. Discussion

P is a vital macronutrient required for plant growth and development, but low P availability is a common nutritional problem in acid soils [3]. Under low-P conditions, most plants generally enhance P absorption efficiency through various strategies to acquire adequate P nutrients [28]. However, for plants that have adapted to acid soils over extended periods of time, maximizing P utilization efficiency may be crucial to sustaining essential life processes. Nevertheless, there are limited studies on the underlying mechanisms of such adaptations. In this study, we investigated tea plants, which exclusively grow in acidic soils, and identified a distinctive adaptive mechanism.

### 3.1. Tea Plant Roots Exhibit Unique Adaptation to Low-P Growing Environments

Plants typically respond to P deficiency by enhancing P absorption efficiency through changes in root architecture, such as increasing root surface area and availability, as reported in previous studies [2,29,30]. However, our investigation found that there was no significant difference in the total root length and number in tea plants treated with different P concentrations (Figure 1B), consistent with prior findings [16]. Interestingly, tea plants do not increase root surface area in response to low-P conditions, as observed in other plant species. Notably, under 0P treatment, the roots of tea plants were healthy, white, and strong. However, the 250P treatment inhibited primary root growth, promoted lateral root development, and resulted in a blunted and senescent phenotype, indicating a unique adaptation mechanism to low P in tea plants. Furthermore, our analysis revealed that the P concentration in tea plant roots reached 16.89 mg·g^−1^ at 250P, surpassing that of other crops, which typically maintain P concentrations of less than 10 mg·g^−1^ even with P treatment concentrations exceeding 500 µmol·L^−1^ [31,32]. This suggests that tea plants have high P utilization efficiency under low-P conditions, which enables normal root growth under 0P treatment. However, under high-P conditions, tea plants exhibit luxury P absorption, inhibiting root growth under 250P treatment. Interestingly, the concentration of Al is promoted by P addition in tea plant roots (Figure 2), which is contrary to common plants. Generally, P is regarded as alleviating Al toxicity by reducing Al uptake in the root [3]. Our previous study reported that Al is an essential element for tea plant root growth [15]. Thus, there must be specific relationships between P and Al in the tea plant, which we need to study further.

Apart from root architecture changes, plants may also regulate the expression of downstream genes such as *PT*, encoded by the phosphate transport protein, through upstream signal networks such as *PHO* and *SPX* to enhance root P transport capacity under low-P conditions [23,33]. Our analysis of P signal network genes in tea plant roots revealed significant regulation of *CsPHO2*, *CsSPX1/2/3/4*, and *CsPT1/2* at different P levels (Figure 3). The regulation pattern of P on *CsPHO2* and *CsSPX1/2/3/4* is consistent with most plants, with low P inhibiting *CsPHO2* expression and promoting the upregulation of *CsSPX1/2/3/4* [23]. However, the expression pattern of *CsPT1/2* in tea plants is opposite to that of *AtPT1.4* in Arabidopsis, with low expression at the 0P condition and significantly enhanced expression at the 250P condition (Figure 3). This suggests that tea plants exhibit high expression of *PT* under high-P conditions, which may contribute to root toxicity caused by excessive P absorption under 250P treatment.

**Figure 6:**
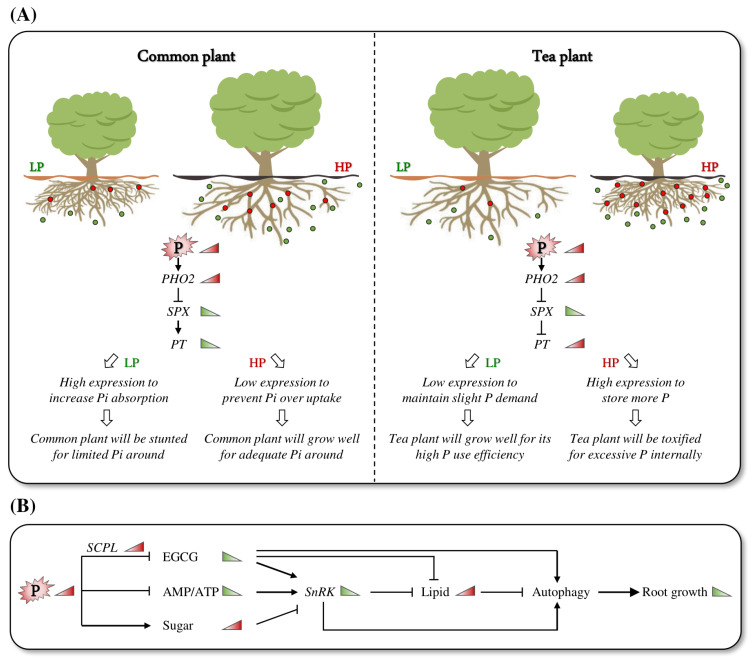
A proposed model illustrating the strong adaptability of tea plant roots to low-P conditions. (**A**) Strategies for plants adaptation to various soil conditions. Common plants are preferred in environments containing adequate P. Under low-P conditions, especially in acid soils, common plants are evolved to increase the expression of *PT* genes to enhance the uptake of Pi. However, the root growth of common plants will be stunted by a prolonged low-P condition. On the contrary, tea plants are well adapted to acid soils with low-P conditions and have a high P utilization efficiency. When there is high P around, tea plants increase *PT* expression to store more Pi. However, excessive P internally could toxify tea plant roots and lead to stunted roots. (**B**) Schematic diagram of P affecting tea plant root growth through the regulation of energy metabolism. A red or green triangle indicates that gene expression or metabolite accumulation is higher or lower under high-P conditions, respectively.

In summary, tea plants have evolved a unique biological mechanism to adapt to low P availability in acid soils. When P is deficient, tea plants rely on high P utilization efficiency to sustain normal root growth, while under high-P conditions, tea plants are prone to P toxicity, negatively affecting growth. Tea plants rapidly enhance *PT* gene expression in response to P signals, which plays a specific biological role in the evolutionary adaptation of tea plants to acid soils (Figure 6A).

### 3.2. Adaptation of Tea Plants to Low P May Be Primarily Regulated by Energy Metabolism

This study revealed the presence of a P regulatory network in tea plant roots, with the energy receptor SnRK1 serving as the core. The network linked energy-related metabolites such as lipids, carbohydrates, and adenosine phosphate to catechins, the main secondary metabolite of tea plants, and cooperatively affected root growth and development (Figure 6B).

The metabolomics analysis showed that 250P treatment significantly increased the accumulation of energy metabolites, such as lipids, carbohydrates, and adenosine, and reduced the AMP/ATP ratio (Figure 5), indicating that the root of tea plants was in a high-energy state under 250P. AMPK is an energy receptor in animals that inhibits gene expression and enzyme activity under high-energy conditions [34]. SnRK1 in plants and Snf1 in yeast are highly homologous to AMPK and regulate cellular metabolism and growth by sensing changes in intracellular energy status [35,36,37]. The analysis of significantly differentially expressed *SnRKs* in the root of tea plants revealed that the expression of *CsSnRK1/2/3* was significantly inhibited by 250P (Figure 5). Therefore, it is speculated that the root of tea plants is in a high-energy state under 250P, resulting in the inhibition of the expression of *SnRKs*, which causes the inhibition of root growth.

Apart from the AMP/ATP ratio, energy sensors can also be inactivated by sensing changes in sugars through phosphorylation and dephosphorylation [34,38]. Trehalose-6-phosphate inhibits SnRK1 activity under high-energy conditions [39]. In addition, EGCG, a unique metabolite of tea plants, induces phosphorylation and activation of AMPK in multiple cell lines [40]. This study found that under 250P treatment, tea plant roots accumulated a large amount of carbohydrate metabolites, including trehalose-6-phosphate, and inhibited the formation of EGCG (Figure 4 and Figure 5; Table S1). Therefore, the ratio of AMP/ATP and the accumulation of EGCG in tea plant roots were decreased under 250P treatment, and the synthesis of carbohydrate compounds was promoted, resulting in the inhibition of the expression of *CsSnRK1/2/3*. AMPK triggers autophagy by phosphorylating the ULK1 protein or inhibiting TORC1 (TOR complex 1) activity, as demonstrated by Kim et al. [41]. Autophagy is a eukaryotic cellular process of macromolecular material degradation and reuse that is essential for maintaining cellular nutrient balance, providing an energy source, and providing a material basis [42]. ATG1 (Autophagy-related gene 1) is a key protein involved in plant autophagy [43]. KIN10, the α subunit of SnRK1, affects autophagy by modulating the phosphorylation of ATG1. Overexpression of KIN10 can delay Arabidopsis leaf senescence and improve tolerance to nitrogen deficiency and other stresses [44]. Moreover, AMPK affects autophagy through indirect pathways. It not only inhibits lipid formation but also promotes its degradation [45]. Knocking out *Snf* in *Mucor circinelloides* can significantly increase lipid content in cells [46]. Phosphatidic acid can also competitively inhibit autophagy [47]. In this study, we observed that the decreased expression of *CsSnRKs* in tea plants led to a substantial accumulation of lipid metabolites under the 250P condition (Figure 5A,B). Thus, we predicted that 250P might inhibit autophagy by reducing *CsSnRKs* expression and increasing lipid accumulation. Furthermore, previous studies have shown that EGCG can promote autophagy. In liver cancer cells, EGCG can inhibit hepatitis B virus replication by inducing autophagy [48]. In conclusion, the reduction in *SnRKs* expression in tea plant roots caused by 250P treatment may lead to a reduction in autophagy and subsequently inhibit the growth of tea plant roots.

## 4. Materials and Methods

### 4.1. Plant Growth

The tea plants utilized in this study were *Camellia sinensis* var. Tieguanyin annual seedlings. The fine roots were absolutely removed before treatments. Subsequently, the seedlings were cultured in a tea nutrient solution for 30 d containing either 0 µmol·L^−1^ or 250 µmol·L^−1^ P. The nutrient solution was maintained at a pH of 4.5 and composed of 400 µmol·L^−1^ AlCl_3_, 250.0 µmol·L^−1^ (NH_4_)_2_SO_4_, 125.0 µmol·L^−1^ K_2_SO_4_, 100.0 µmol·L^−1^ MgSO_4_, 62.5 µmol·L^−1^ Ca(NO_3_)_2_, 37.5 µmol·L^−1^ CaCl_2_, 25.3 µmol·L^−1^ KH_2_PO_4_, 16.4 µmol·L^−1^ Fe(III)-EDTA, and slight amounts of MnSO_4_, H_3_BO_3_, ZnSO_4_, CuSO_4_, and (NH_4_)_6_Mo_7_O_24_ [15]. There were five biological replicates for phenotypic analysis and three replicates for ionomic, metabolomic, and transcriptomic analysis, respectively. After different P treatments, the roots were pictured immediately for phenotypic analysis, dried for ionomic analysis, and stored at −80 °C for metabolomic, transcriptomic, and gene expression analysis. The experiments were conducted in an artificial climate growth room with a light/dark time of 14/10 h, a light intensity of 200 μmol m^−2^ s^−1^, and a temperature of 27 °C.

### 4.2. Phenotypic Analysis

After P treatments, the roots were photographed using a digital camera (Nikon Company, Minato City, Japan). The newly grown roots were then scanned and analyzed using the WinRHIZO LA2400 root system (Regent Company, Richmond, BC, Canada) for total root length. The main root length was measured via Image J software (version 1.52a).

### 4.3. Ionomic Analysis

After treatments, the newly grown roots were harvested, rinsed with distilled water three times, and oven-dried at 105 °C for 30 min, followed by further drying at 70 °C until a constant weight was achieved. After being ground into powder, 0.010 g of the sample was weighed and heated gradually to 120 °C in concentrated HNO_3_ for complete digestion. After cooling, the solution was added to a volume of 20 mL. Furthermore, 1 mL of digestive solution was diluted about four-fold using ultrapure water. Finally, the nutrient element quantification was performed using an inductively coupled plasma mass spectrometer, the ICP-MS7900 (Agilent Company, Santa Clara, CA, USA). Sample powder (0.010 g) was digested in nitric acid and heated gradually to 120 °C, and nutrient quantification was performed using an inductively coupled plasma mass spectrometer 7900 (Agilent Company, Santa Clara, CA, USA).

### 4.4. Metabolomic Analysis

The root samples were collected and detected for metabolomes by Metware Biotechnology Co., Ltd. (Wuhan, China). For metabolomic analysis, the freeze-dried sample was crushed and extracted with methanol, and the supernatant was retained for analysis. The supernatant was detected with the SB-C1 1.8 µm, 2.1 mm × 100 mm liquid chromatographic column (Agilent Company, Santa Clara, CA, USA) and analyzed by the AB4500 Q TRAP UPLC-ESI-MS/MS system (Shimadzu Company, Kyoto, Japan) in the ESI-triple quadrupole linear ion trap (QTRAP) mass spectrometer. The mobile phase is composed of solvent A (water with 0.1% formic acid) and solvent B (acetonitrile with 0.1% formic acid). The sample measurement was conducted with a gradient program. The flow rate is 0.35 mL/min^−1^. The column temperature is about 40 °C. The mass spectrometer is equipped with an ESI Turbo Ion-Spray interface, which allows operation in both positive and negative ion modes. The collected information was qualitatively analyzed on the basis of the Metware database, and the peak area of each chromatographic peak represented the relative content of the substance. Principal component analysis and cluster analysis were conducted on all samples, and differentially accumulated metabolites were screened based on the criteria of *p* < 0.05 and log_2_FC (fold change) > 1.

### 4.5. Transcriptomic Analysis

The root samples were also analyzed by RNA-seq by Metware Biotechnology Co., Ltd. (Wuhan, China). Total RNA was extracted from the roots using the TRIzol reagent (Invitrogen Company, Waltham, MA, USA), and the RNAseq library was prepared using the NEBNext Ultra RNA library kit (New England Biolabs Company, Ipswich, MA, USA). The sequencing data were generated on the Illumina platform, and the raw data were filtered and aligned to the Tieguanyin genome (https://ngdc.cncb.ac.cn/gwh/Assembly/12834/show accessed on 1 October 2022). The gene expression levels were calculated based on the FPKM value, and bioinformatics analyses were performed to screen for differential gene expression and conduct functional annotation based on the expression levels of genes in different samples. Differential gene screening was conducted using a threshold of *p*adj < 0.05 and an absolute log_2_FC (fold change) > 1.

### 4.6. Gene Expression Analysis

For the verification of gene expression, total RNA was extracted from the root samples using the CTAB method [49]. Furthermore, 500 ng of total RNA was reverse transcribed to cDNA using the PrimeScript kit (Takara Company, Kusatsu, Japan). The PCR cycling was 95 °C for 1 min, 40 cycles of 95 °C for 15 s and 58 °C for 15 s, and 72 °C for 30 s through light cycler 96 (Roche Company, Basel, Switzerland). The expression of the *CsActin* gene was used as the internal reference for calculating the relative gene expression level (Table S4).

### 4.7. Evolutionary Tree Analysis

The amino acid sequences of homologous genes in rice, soybean, and Arabidopsis were obtained from online databases, including MSU-RGAP (http://rice.uga.edu/index.shtml accessed on 1 November 2022), Phytozome (https://phytozome-next.jgi.doe.gov/ accessed on 1 November 2022), and Tair (https://www.arabidopsis.org/ accessed on 13 November 2022). The amino acid sequences were aligned, and an evolutionary tree was constructed using the Neighbor Joining (NJ) method and Poisson model in MEGA software (version 10.2.6).

### 4.8. Statistical Analysis

The data were analyzed by *t*-tests with SPSS software (version 19.0).

## 5. Conclusions

The tea plants adapt well to acid soil with limited available phosphate. Our study revealed that the tea plant roots remained healthy under 0P, while they suffered under 250P, likely due to their high P utilization efficiency under 0P and over-uptake of phosphate under high-P conditions.

Tea plants have unique mechanisms to adapt to low-P conditions in acid soils. However, excessive P application can inhibit the growth of tea plant roots and cause P toxicity. In this study, we found that high P (250P) inhibited the elongation of tea plant roots and caused P toxicity, which was possibly due to the excessive expression of phosphate uptake-responsible genes (*CsPT1/2*). Moreover, P over-accumulation under 250P conditions regulates energy metabolism and thus root growth. Under low-P conditions in acid soils, tea plants regulate the synthesis and accumulation of energy-related metabolites such as lipids, carbohydrates, and adenosine by controlling the expression and activation of the energy-sensing kinase CsSnRKs. In the aspect of tea quality, high P could also inhibit the esterification of catechins in the tea plant roots, which might be carried out by decreasing the expression of esterification genes, such as *CsTGY09G0002412b*, and finally reducing the accumulation of EGCG. These findings suggest that tea plants have developed a delicate balance in P uptake and utilization during the evolution of adaptation to low-P conditions. In the management of tea plantations, excessive P fertilizer application should be avoided to ensure the promotion of healthy root growth and tea quality.

## Data Availability

The data that supports the findings of this study is available in the Supplementary Materials of this article.

## Supplementary Materials

The following supporting information can be downloaded at: https://www.mdpi.com/article/10.3390/ijms241512431/s1.
