# Supplementary material for: Multi-Omics Analysis Reveals Mechanisms of Strong Phosphorus Adaptation in Tea Plant Roots
Source: Int J Mol Sci. 2023 Aug 4;24(15):12431. doi: 10.3390/ijms241512431 (PMC10419353; doi:10.3390/ijms241512431)

Table S1. Differentially changed P-contained metabolites affected by P treatments through metabolomics. The red number indicates the accumulation of compound was increased by 250P, while the green number shows that was decreased.

NO.	Compounds	Formula	Ionization model	Molecular weight (Da)	*P*	VIP	Peak area × 10^3 (Means±Standard error)		Log₂ (250P /0P)
							0P	250P	
**Lipids**									
L1	LysoPC 18:4	C26H46NO7P	[M+H]+	515.30	0.00	1.34	0.01±0.00	121.73±5.23	**13.72**
L2	LysoPE 18:4	C23H40NO7P	[M+H]+	473.25	0.00	1.34	0.01±0.00	15.49±0.75	**10.75**
L3	LysoPE 20:5	C25H42NO7P	[M+H]+	499.27	0.01	1.34	0.01±0.00	14.94±1.57	**10.70**
L4	LysoPE 16:3	C21H38NO7P	[M+H]+	447.24	0.01	1.34	0.01±0.00	3.74±0.33	**8.70**
L5	LysoPC 17:0	C25H52NO7P	[M+H]+	509.35	0.00	1.32	123.46±29.16	3895.07±68.44	**4.98**
L6	LysoPC 17:0(2n isomer)	C25H52NO7P	[M+H]+	509.35	0.00	1.32	123.46±29.16	3895.07±68.44	**4.98**
L7	LysoPE 16:0	C21H44NO7P	[M+H]+	453.29	0.00	1.34	436.55±9.23	11623.33±472.23	**4.73**
L8	LysoPC 18:0(2n isomer)	C26H54NO7P	[M+H]+	523.36	0.01	1.32	304.19±68.10	6585.43±611.04	**4.44**
L9	LysoPE 18:0	C23H48NO7P	[M+H]+	481.32	0.00	1.34	11.56±0.33	246.90±7.79	**4.42**
L10	LysoPC 16:0	C24H50NO7P	[M+H]+	495.33	0.00	1.34	4602.47±99.71	85037.67±1359	**4.21**
L11	LysoPC 20:5	C28H48NO7P	[M+H]+	541.32	0.00	1.34	10.15±0.30	172.19±7.63	**4.08**
L12	LysoPC 16:2	C24H46NO7P	[M+H]+	491.30	0.02	1.07	5.33±5.32	85.67±14.71	**4.01**
L13	LysoPC 20:2	C28H54NO7P	[M+H]+	547.36	0.02	1.32	16.80±2.60	202.75±25.02	**3.59**
L14	LysoPC 20:3	C28H52NO7P	[M+H]+	545.35	0.01	1.33	76.53±1.81	610.46±49.49	**3.00**
L15	Choline alfoscerate	C8H20NO6P	[M+H]+	257.10	0.01	1.33	414.74±17.5	2847.83±253.07	**2.78**
L16	LysoPE 20:3(2n isomer)	C25H46NO7P	[M+H]+	503.30	0.01	1.33	5.92±0.14	37.53±3.15	**2.66**
L17	LysoPE 20:3	C25H46NO7P	[M+H]+	503.30	0.00	1.33	5.78±0.20	35.45±1.77	**2.62**
L18	LysoPC 15:1	C23H46NO7P	[M+H]+	479.30	0.00	1.32	39.36±5.66	237.56±7.69	**2.59**
L19	LysoPC 16:2(2n isomer)	C24H46NO7P	[M+H]+	491.30	0.00	1.34	70.53±0.60	383.10±13.41	**2.44**
L20	LysoPC 20:1	C28H56NO7P	[M+H]+	549.38	0.00	1.33	85.43±5.36	453.25±13.55	**2.41**
L21	LysoPC 20:4	C28H50NO7P	[M+H]+	543.33	0.00	1.33	266.97±9.01	1380.83±65.25	**2.37**
L22	LysoPE 18:3(2n isomer)	C23H42NO7P	[M+H]+	475.27	0.00	1.34	4909.70±125.21	23514.00±527.77	**2.26**
L23	LysoPE 20:4(2n isomer)	C25H44NO7P	[M+H]+	501.29	0.00	1.33	32.61±1.85	150.40±5.04	**2.21**
L24	LysoPC 16:1	C24H48NO7P	[M+H]+	493.32	0.00	1.23	1429.65±377.74	6103.40±233.11	**2.09**
L25	LysoPC 19:1	C27H54NO7P	[M+H]+	535.36	0.00	1.33	646.27±27.79	2519.43±52.54	**1.96**
L26	LysoPC 22:4	C30H54NO7P	[M+H]+	571.36	0.02	1.32	13.27±0.51	49.58±4.85	**1.90**
L27	LysoPC 18:3(2n isomer)	C26H48NO7P	[M+H]+	517.32	0.00	1.34	1805.80±29.06	6178.17±223.6	**1.77**
L28	LysoPC 18:3	C26H48NO7P	[M+H]+	517.32	0.00	1.34	1805.80±29.06	6178.17±223.6	**1.77**
L29	LysoPC 16:1(2n isomer)	C24H48NO7P	[M+H]+	493.32	0.01	1.33	1638.70±33.58	5516.23±305.68	**1.75**
L30	LysoPC 17:2	C25H48NO7P	[M+H]+	505.32	0.00	1.33	125.10±7.09	377.84±6.42	**1.59**
L31	LysoPE 15:0(2n isomer)	C20H42NO7P	[M+H]+	439.27	0.00	1.33	940.54±12.65	2694.37±88.83	**1.52**
L32	LysoPE 16:1(2n isomer)	C21H42NO7P	[M+H]+	451.27	0.00	1.31	4364.60±465.98	12168.00±189.96	**1.48**
L33	LysoPE 18:2(2n isomer)	C23H44NO7P	[M+H]+	477.29	0.00	1.34	474.33±4.42	1297.47±40.81	**1.45**
L34	LysoPC 15:0(2n isomer)	C23H48NO7P	[M+H]+	481.32	0.04	1.26	116.77±13.02	318.80±45.78	**1.45**
L35	LysoPC 20:2(2n isomer)	C28H54NO7P	[M+H]+	547.36	0.00	1.34	49.73±0.68	133.86±3.61	**1.43**
L36	LysoPE 18:0(2n isomer)	C23H48NO7P	[M+H]+	481.32	0.00	1.33	40.04±1.96	106.47±3.27	**1.41**
L37	LysoPE 18:1(2n isomer)	C23H46NO7P	[M+H]+	479.30	0.00	1.33	5078.43±126.28	13195.67±480.67	**1.38**
L38	LysoPC 16:0(2n isomer)	C24H50NO7P	[M+H]+	495.33	0.02	1.32	968.45±8.59	2509.60±200.54	**1.37**
L39	LysoPE 18:1	C23H46NO7P	[M+H]+	479.30	0.00	1.33	4837.00±110.1	12477.67±457.67	**1.37**
L40	LysoPE 15:1(2n isomer)	C20H40NO7P	[M+H]+	437.25	0.00	1.31	25.62±2.31	64.27±1.38	**1.33**
L41	LysoPC 22:5	C30H52NO7P	[M+H]+	569.35	0.01	1.18	30.77±6.38	73.23±4.45	**1.25**
L42	LysoPC 19:2	C27H52NO7P	[M+H]+	533.35	0.00	1.24	36.57±5.82	86.78±6.27	**1.25**
L43	LysoPE 16:1	C21H42NO7P	[M+H]+	451.27	0.00	1.33	5260.53±35.20	12005.33±333.38	**1.19**
L44	LysoPE 15:1	C20H40NO7P	[M+H]+	437.25	0.00	1.34	171.09±3.21	372.37±5.52	**1.12**
L45	LysoPC 19:2(2n isomer)	C27H52NO7P	[M+H]+	533.35	0.01	1.31	102.81±4.29	212.87±14.18	**1.05**
L46	LysoPC 18:2	C26H50NO7P	[M+H]+	519.33	0.00	1.33	870.71±3.96	1767.77±50.36	**1.02**
L47	LysoPC 18:2(2n isomer)	C26H50NO7P	[M+H]+	519.33	0.00	1.33	870.71±3.96	1767.77±50.36	**1.02**
L48	LysoPC 18:1(2n isomer)	C26H52NO7P	[M+H]+	521.35	0.00	1.33	6393.00±56.69	12928.00±283.04	**1.02**
L49	LysoPE 14:0(2n isomer)	C19H40NO7P	[M+H]+	425.25	0.00	1.33	85.32±1.45	172.12±5.22	**1.01**
L50	LysoPC 16:4	C24H42NO7P	[M+H]+	487.27	0.00	1.34	181.62±1.06	17.69±1.14	**-3.36**
L51	LysoPE 17:1	C22H44NO7P	[M+H]+	465.29	0.00	1.34	2037.10±19.32	0.01±0.00	**-17.79**

(continued)

NO.	Compounds	Formula	Ionization model	Molecular weight (Da)	*P*	VIP	Peak area × 10^3 (Means±Standard error) 0P	250P	Log_2 (250P /0P)
**Nucleotides and derivatives**									
ND1	Uridine 5'-monophosphate	C9H13N2O9P	[M-H]-	324.04	0.00	1.33	21.78±2.34	183.66±10.02	**3.08**
ND2	2-Deoxyribose-1-phosphate	C5H11O7P	[M-H]-	214.02	0.00	1.30	23.70±4.15	126.07±6.57	**2.41**
ND3	5-Aminoimidazole ribonucleotide	C8H14N3O7P	[M+H]+	295.06	0.01	1.33	524.10±11.38	2272.07±204.85	**2.12**
ND4	NADP (Nicotinamide adenine dinucleotide phosphate)	C21H28N7O17P3	[M-H]-	743.08	0.02	1.31	56.28±3.14	239.59±28.39	**2.09**
ND5	Uridine 5'-diphospho-D-glucose	C15H24N2O17P2	[M-H]-	566.06	0.01	1.33	1868.23±13.98	7739.03±678.67	**2.05**
ND6	Adenosine 5'-monophosphate	C10H14N5O7P	[M+H]+	347.06	0.00	1.31	34.91±5.08	141.97±7.66	**2.02**
ND7	Uridine-5'-diphosphate-D-xylose	C14H22N2O16P2	[M-H]-	536.04	0.00	1.33	219.91±4.29	751.95±29.86	**1.77**
ND8	Uridine 5'-diphosphate	C9H14N2O12P2	[M-H]-	404.00	0.00	1.32	10.26±1.02	33.26±1.33	**1.70**
ND9	Adenosine 5'-diphosphate	C10H15N5O10P2	[M-H]-	427.03	0.04	1.29	40.76±2.98	118.27±15.93	**1.54**
ND10	Uridine 5'-diphospho-N-acetylglucosamine	C17H27N3O17P2	[M-H]-	607.08	0.00	1.26	102.42±17.62	264.95±10.39	**1.37**
ND11	Cyclic 3',5'-adenylic acid	C10H12N5O6P	[M-H]-	329.05	0.00	1.07	26.51±0.94	2.09±2.08	**-3.66**
**Saccharides and alcohols**									
SA1	Dihydroxyacetone phosphate	C3H7O6P	[M-H]-	170.00	0.03	1.34	0.01±0.00	219.62±37.72	**14.57**
SA2	D-Glucose 1,6-bisphosphate	C6H14O12P2	[M-H]-	340.00	0.01	1.34	0.01±0.00	53.64±6.38	**12.54**
SA3	Sorbitol-6-phosphate	C6H15O9P	[M-H]-	262.05	0.01	1.34	0.01±0.00	49.01±3.7	**12.41**
SA4	D-Glucose 6-phosphate*	C6H13O9P	[M-H]-	260.03	0.00	1.34	234.96±9.50	4601.80±62.26	**4.29**
SA5	Glucose-1-phosphate*	C6H13O9P	[M-H]-	260.03	0.00	1.34	206.93±14.74	3977.64±177.86	**4.26**
SA6	D-Fructose 6-phosphate	C6H13O9P	[M-H]-	260.03	0.02	1.33	207.66±14.53	3058.77±441.14	**3.88**
SA7	Trehalose 6-phosphate	C12H23O14P	[M-H]-	422.08	0.02	1.28	22.26±5.30	118.21±15.06	**2.41**
SA8	D-Glucosamine 1-phosphate	C6H14NO8P	[M-H]-	259.05	0.00	1.33	107.85±3.22	452.75±23.5	**2.07**
SA9	D-Erythrose-4-phosphate	C4H9O7P	[M-H]-	200.01	0.04	1.28	289.05±19.14	822.44±110.19	**1.51**
SA10	Glucarate O-phosphoric acid	C6H11PO11	[M-H]-	290.00	0.03	1.29	1365.83±108.78	3752.70±442.51	**1.46**
SA11	D-Sedoheptuiose 7-phosphate	C7H15O10P	[M-H]-	290.04	0.00	1.32	1416.87±93.3	3607.30±148.22	**1.35**
**Organic acids**									
OA1	Phosphoenolpyruvate	C3H5O6P	[M-H]-	167.98	0.00	1.34	0.01±0.00	1303.27±61.40	**17.14**
OA2	2-Hydroxyethylphosphonic acid	C2H7O4P	[M-H]-	126.01	0.01	1.23	62.83±2.51	26.23±4.77	**-1.26**
**Alkaloids**									
A1	O-Phosphorylethanolamine	C2H8NO4P	[M-H]-	141.02	0.00	1.33	11.06±1.81	206.66±8.38	**4.22**
**Others**									
O1	Propyl 2-(trimethylammonio) ethyl phosphate	C28H50NO7P	[M+H]+	543.33	0.00	1.34	374.61±6.28	1347.67±37.3	**1.85**

Table S2. Differentially changed lipid metabolites without P affected by P treatments through metabolomics. The red number indicates the accumulation of compound was increased by 250P, while the green number shows that was decreased.

Compounds	Formula	Ionization model	Molecular weight (Da)	*P*	VIP	Peak area × 10^3 (Means±Standard error) 0P	250P	Log_2 (250P /0P)
**Free fatty acids**								
(E)-Linalool-1-oic acid	C10H16O3	[M-H]-	184.11	0.03	1.34	0.01±0.00	36.81±6.65	**12.00**
1-Linolenoyl-rac-glycerol-diglucoside	C33H56O14	[M+H]+	676.37	0.00	1.29	142.81±31.87	994.91±24.28	**2.80**
Arachidonic acid	C20H32O2	[M-H]-	304.24	0.01	1.31	21.39±3.31	114.44±12.10	**2.42**
13-Hydroxy-9Z,11E-octadecadienoic acid	C19H34O3	[M+H]+	310.25	0.00	1.24	2.17±0.81	11.15±0.30	**2.36**
Cis-10-pentadecenoic acid (C15: 1)	C15H28O2	[M-H]-	240.21	0.00	1.33	9060.40 ±502.90	33254.67 ±830.92	**1.88**
Oleic acid	C18H34O2	[M+H]+	282.26	0.00	1.21	2.43±0.56	7.66±0.53	**1.66**
1-Linoleoyl-sn-glycerol-diglucoside	C33H58O14	[M+H]+	678.38	0.00	1.33	32.67±0.95	94.80±2.23	**1.54**
9,12,13-Trihome	C18H34O5	[M-H]-	330.24	0.00	1.32	66.37±3.62	149.37±3.85	**1.17**
9,10,13-Trihydroxy-11-octadecenoic acid	C18H34O5	[M-H]-	330.24	0.00	1.33	48.02±1.73	103.57±2.12	**1.11**
13(S)-Hode;13(S)-hydroxyoctadeca-9Z,11E-dienoic acid	C18H32O3	[M-H]-	296.24	0.00	1.33	44.72±2.10	91.48±0.38	**1.03**
Ethyl 9-hydroxy-10,12-octadecadienoic acid	C20H36O3	[M+H]+	324.27	0.02	1.32	65.82±7.32	12.60±0.32	**-2.39**
**Glycerol ester**								
1-α-Linolenoyl-glycerol-3-O-glucoside	C27H46O9	[M+H]+	514.31	0.01	1.15	1.87±1.86	107.28±9.43	**5.84**
2-α-Linolenoyl-glycerol-1-O-glucoside	C27H46O9	[M+H]+	514.31	0.03	1.02	3.47±1.88	97.95±16.41	**4.82**
Gingerglycolipid A	C33H56O14	[M-H]-	676.37	0.01	1.33	64.55±3.07	354.52±22.97	**2.46**
2-α-Linolenoyl-glycerol-1,3-di-O-glucoside	C33H56O14	[M+H]+	676.37	0.01	1.32	13.93±1.49	57.97±4.46	**2.06**
1-α-Linolenoyl-glycerol-2,3-di-O-glucoside	C33H56O14	[M+H]+	676.37	0.03	1.31	14.46±0.17	48.59±5.90	**1.75**
1-Linoleoylglycerol-2,3-di-O-glucoside	C33H58O14	[M+H]+	678.38	0.00	1.32	31.43±2.24	93.01±3.01	**1.57**
1-Oleoyl-sn-glycerol	C21H40O4	[M+H]+	356.29	0.03	1.28	96.16±8.37	277.83±35.46	**1.53**
2-Linoleoylglycerol-1,3-di-O-glucoside	C33H58O14	[M+H]+	678.38	0.00	1.32	35.89±2.63	94.38±4.72	**1.39**
Gingerglycolipid B	C33H58O14	[M-H]-	678.38	0.00	1.32	246.58±13.27	643.82±30.20	**1.38**

Table S3. Differentially changed carbohydrate metabolites without P affected by P treatments through metabolomics. The red number indicates the accumulation of compound was increased by 250P, while the green number shows that was decreased.

Compounds	Formula	Ionization model	Molecular weight (Da)	*P*	VIP	Peak area × 10^3 (Means±Standard error)		Log_2 (250P/0P)
						0P	250P	
1,6-Anhydro-β-D-glucose	C6H10O5	[M-H]-	162.05	0.01	1.34	0.01±0.00	22.34±2.62	**11.28**
Maltotriose	C18H32O16	[M+Na]+	504.17	0.00	1.32	105.34±8.79	300.06±2.17	**1.51**
D-Xylonic acid	C5H10O6	[M-H]-	166.05	0.01	1.31	862.55±56.55	399.59±20.22	**-1.11**
Dmelezitose O-rhamnoside	C24H42O20	[M-H]-	650.23	0.00	1.30	948.60±51.73	403.93±33.31	**-1.23**

Table S4. Primer sequences.

Gene names	Primer sequences
CsPHO2	F: GTTGAGCACTGCCGTGATATTGC<br>R: CCCAAAGCCAAACCATCATAGTC
CsSPX1	F: AGAGGACTTTGTGTACTTGTTGGA<br>R: CTGTAATTCATCAAGAGCACCATTT
CsSPX2	F: GGCTCCTCCGATGATGAATGG<br>R: ACCCATCTCCTCTTTGTAATCCA
CsSPX3/4	F: GCCTGCCAAGCGCCCTAG<br>R: CTTTATCATCTCTTCACTCCAATCT
CsPT1/2	F: GAGCAGCAGAAAGTAGACCAGATA<br>R: GCCCTTGCTATTCTAAAGACTTCAT
CsTGY09G0002412b	F: CTCTGCCTTCTCTGCTTTCATTTAT<br>R: CGGTAGATTTAGAAGCAAGAGAGGT
CsTGY01G0000352a	F: GCTGCACTGATTGGTTCCAC<br>R: TTCTTCCCCTTCGCTCCAG
CsSnRK1	F: AAGAGATCACAAGTTTCGAGGAC<br>R: TGCAGCCTCAAACTGGAGT
CsSnRK2	F: GAAGCGCAGAAAACTGGGCAC<br>R: CAAGATGACGATGATGACGCAG
CsSnRK3	F: AGTGATGCAGTCAAGATTTTATCCG<br>R: CCGAATGCCACTGCTCCGA
CsActin	F: GGCAGATAGATGCTTATGTAGGTG<br>R: TGTTTGCTTTAGGGTTGAGTGG

Figure S1. Phylogenetic trees of CsSPXs, CsPHOs and CsPTs. (A) SPXs. (B) PHOs. (C) PTs. At, arabidopsis; Os, rice; Gm, soybean. Red triangle indicates the key genes associated with P signal network in tea plant roots affected by P treatments.

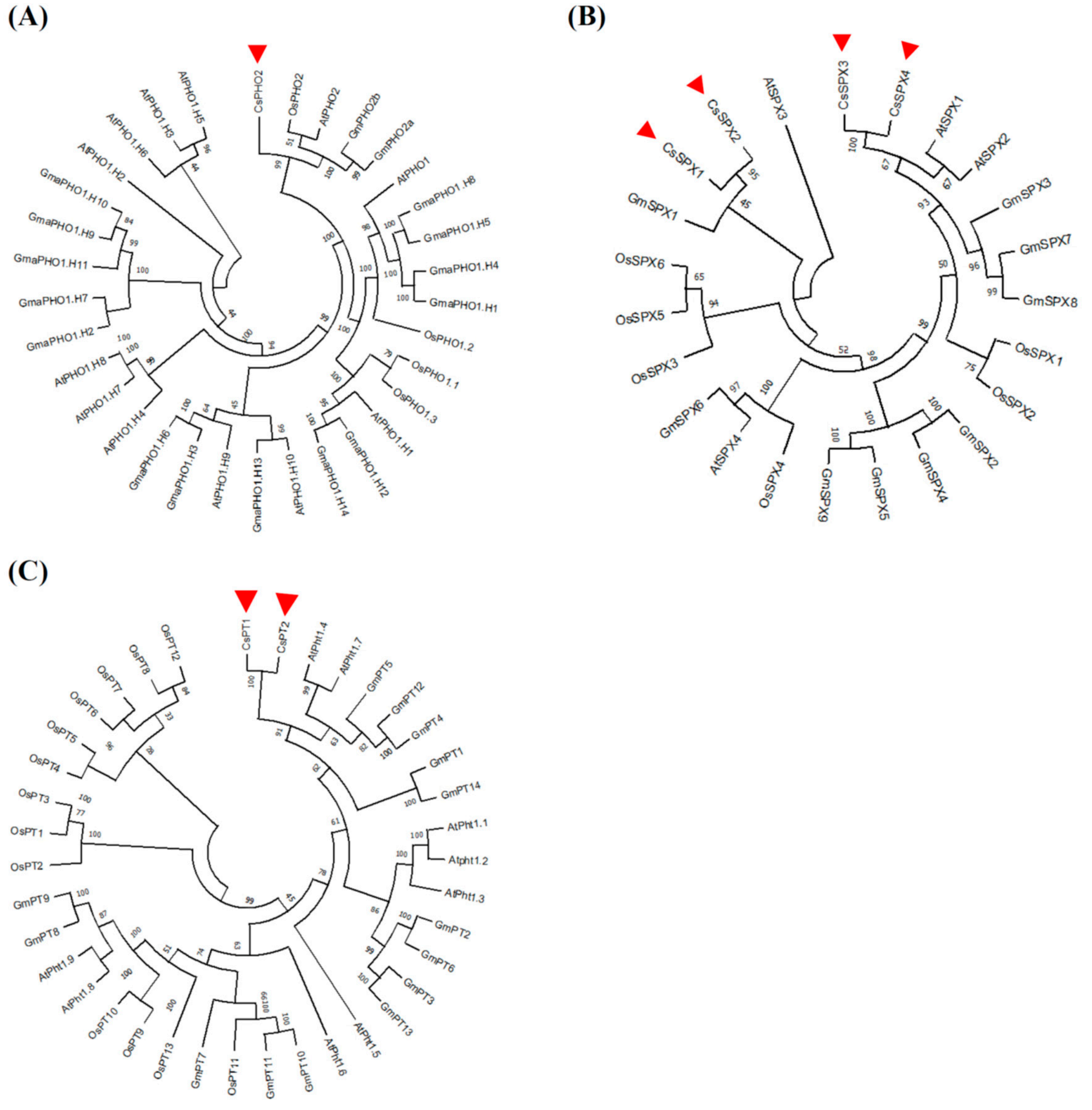

Figure S2. Relative expression of key genes in the roots of tea plants in response to P treatments. *Actin* was used as an internal standard. 0P, 0 μmol·L^{-1} P; 250P, 250 μmol·L^{-1} P. Three biological replicates were performed for each treatment. Asterisks indicate statistical differences. ns, no statistical differences; *, significant differences at 0.01 < P ≤ 0.05; **, significant differences at 0.001 < P ≤ 0.01; ***, significant differences at P ≤ 0.001.

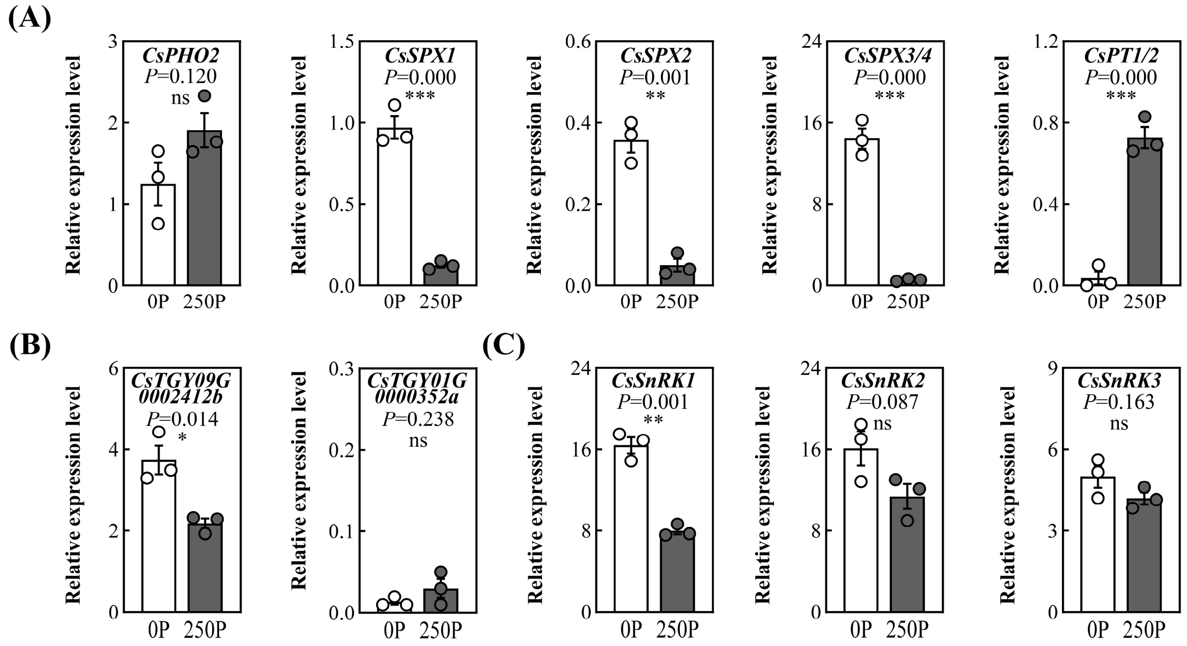

Supplement: Supplementary file 1 [file ijms-24-12431-s001.zip › ijms-2483610-supplementary.pdf]
